# Supplementary material for: Giant enhancement of THz-frequency optical nonlinearity by phonon polariton in ionic crystals
Source: Nat Commun. 2021 May 26;12:3183. doi: 10.1038/s41467-021-23526-w (PMC8155090; doi:10.1038/s41467-021-23526-w)
Supplement: Supplementary file 1 — Supplementary Information [file 41467_2021_23526_MOESM1_ESM.pdf]

Supplementary Information for

Giant enhancement of THz-frequency optical nonlinearity by phonon polariton in ionic crystals

Yao Lu *et al.*

Supplementary Note 1: Calculation of the experimental second-order nonlinear susceptibility

The second-order nonlinear susceptibility is calculated based on the coupled-wave equations[1] by considering two special aspects in our system: (I) the mode dispersion of the LN waveguide[2] causes the major difference. On one hand, the THz waves generated by the difference frequency $\nu_\mathrm{d} = \nu_1 - \nu_0$ are not the eigenmodes, so they are not supported by the LN waveguide. On the other hand, the mode dispersion also causes a walk-off effect in time between THz waves with frequencies ν_0 and ν_1; (II) The absorption of THz waves by the LN material also exerts some influence.

The walk-off effect is caused by the difference in THz group velocities with frequencies ν_0 and ν_1, which does not change during the DFG process. Therefore, this effect can be described by simply setting a parameter $p = 1 - x/x_\mathrm{end}$, where $x_\mathrm{end} = 2.2$ mm is the position where the walk-off effect is finished and DFG signal stops generating, whose value is determined by the experiments.

As the DFG signal is not supported by the LN waveguide, the mode attenuation can be calculated by analyzing the phase change during its propagation. Since the THz waves with frequencies ν_0 and ν_1 are the zero and first order TE modes of the waveguide, the following expression can be obtained[2]

$$\begin{aligned} \kappa_0 d &= \phi, \\ \kappa_1 d &= \phi + \pi, \end{aligned} \tag{S1}$$

where κ_0 and κ_1 are the wavevectors orthogonal to propagation direction for the two modes, and ϕ indicates the phase-shift in the mode equation of the waveguides. In Eq. (S1), the difference of the phase-shift in different modes is ignored.

According to the phase-matching condition, the propagation constants of the THz waves satisfy the condition $\beta_\mathrm{d} = \beta_1 - \beta_0$, therefore $\kappa_i \propto \nu_i$ with i =1, 2, and d. Thus, the phase-change $\varphi = -2\phi = 0.92\pi$ rad can be easily calculated, and the corresponding loss per millimeter is $\Gamma_M E_\mathrm{d} = 20.43 E_d$, where E_d is the electric field of THz waves with frequency ν_d. At the same time, THz waves are also absorbed by LN. At room temperature, the absorption coefficients for the THz waves with frequencies of ν_0, ν_1, and ν_d are $\alpha_0 = 0.3$ mm^{-1}, $\alpha_1 = 1.5$ mm^{-1}, and $\alpha_\mathrm{d} = 0.9$ mm^{-1}, respectively[3].

It is assumed that the THz electric fields for the three frequencies at the position x are $E_0(x)$, $E_1(x)$, and $E_\mathrm{d}(x)$, respectively. Considering the change in the E-field from position x to $x + dx$, we can write the change in the field E_0 and E_1, which mainly comes from the generation of femtosecond laser and the material absorption, as follows:

$$\begin{aligned} dE_0 &= I_{G0} \exp(-\Gamma|x-d|)\, dx - \alpha_0 E_0 dx, \\ dE_1 &= I_{G1} \exp(-\Gamma|x-d|)\, dx - \alpha_1 E_1 dx, \end{aligned} \tag{S2}$$

where I_{G0} and I_{G1} depend on the effective intensity of the laser pulse due to the properties of impulsive stimulated Raman scattering[4,5]. The function $\exp(-\Gamma|x-d|)$ with parameters Γ and d represents the power change of the pump laser pulses. Here, we assume $0 < d < x_1$ according to the experimental results.

According to the initial value of $E_0(x=0) = E_1(x=0) = 0$, $E_0(x)$ and $E_1(x)$ can be analytically solved as

$$E_0 = e^{-\alpha_0 x}\int_0^x I_{G0}\, e^{\alpha_0\xi-\Gamma|\xi-d|}d\xi,$$
$$E_1 = e^{-\alpha_1 x}\int_0^x I_{G1}\, e^{\alpha_1\xi-\Gamma|\xi-d|}d\xi, \tag{S3}$$

By fitting all the spectra of THz waves as in Fig. 4b, the electric fields of THz waves at different frequencies and different positions can be obtained, as shown in Supplementary Table 1. Using the same method as what we used in Fig. 5, we can get the boundary condition to evaluate the fitting parameter in Eq. (S3).

Supplementary Table 1 The electric fields of THz waves at different frequencies and different positions.

	E_0 (kV/mm)	E_1 (kV/mm)	E_d (kV/mm)
x_1	3.29	1.71	0.935
x_2	3.659	1.26	0.453
x_3	3.67	0.61	---
x_4	2.74	0.24	---

We solve for the values of the parameters and obtain that

$$I_{G0} = 4.70 \text{ kV/mm}^2;$$
$$I_{G1} = 4.39 \text{ kV/mm}^2;$$
$$\Gamma = 1.28 \text{ mm}^{-1};$$
$$d = 0.650 \text{ mm}; \tag{S4}$$

The change in E_d signal is mainly due to the DFG, material absorption, and the waveguide mode attenuation. Thus

$$dE_d = E_{DFG} - \alpha_d E_d dx - \Gamma_M E_d dx, \tag{S5}$$

$$E_{\mathrm{DFG}} = p\chi^{(2)} \frac{2\pi\nu_{\mathrm{d}} E_0 E_1}{c n_{\mathrm{eff}}} dx,$$

where E_{DFG} stands for the DFG of the field E_{d} signal in terms of E_0 and E_1. Here, we think the contribution of pump laser generation at the DFG frequency is ignorable, and it makes little difference to our results. The initial and boundary conditions are considered to be

$$E_{\mathrm{d}}(x = 0) = 0;$$
$$E_{\mathrm{d}}(x = 1.18) = 0.935 \text{ kV/mm}. \quad \text{(S6)}$$

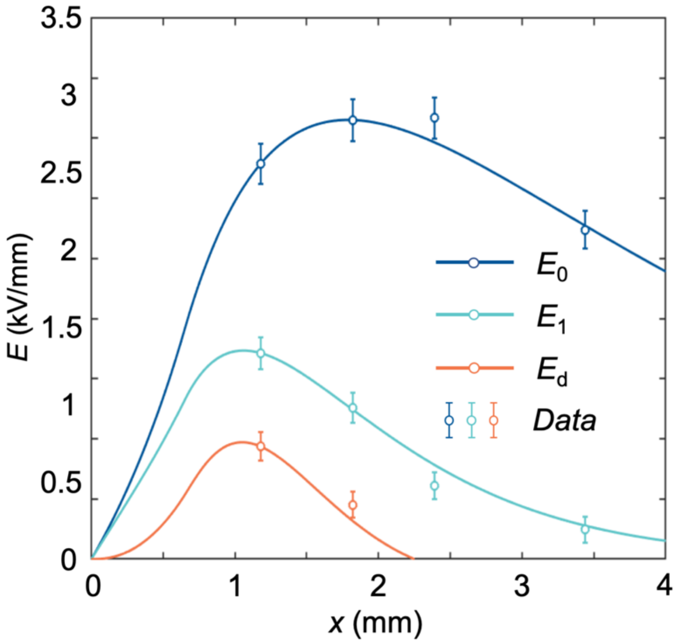

Supplementary Figure 1 Plot of the electric field of THz waves as a function of position x. The solid curves show calculated results and the circles are from experimental results. The error bar is evaluated from the fitting deviation and the experimental noise. E_0, E_1, and E_{d} are the electric field amplitudes of THz waves with frequency ν_0, ν_1, and ν_{d}, respectively.

The units used above are millimeter (mm), kilovolt (kV), and picosecond (ps). Substituting E_0 and E_1 in Eq. (S5) with Eq. (S3) and the parameters in Eq. (S4), we can solve Eq. (S5). The value of the second-order nonlinear susceptibility can be obtained by using the boundary condition Eq. (S6), and the result is $\chi^{(2)}$ =1.58 mm/kV= 1.58×10^{-6} m/V.

Substituting Eq. (S5) with this result, we get the solution of $E_{\mathrm{d}}(x)$. The corresponding electric fields dependent on position x are shown in Supplementary Fig. 1. Three solid curves show the calculated results of $E_0(x)$, $E_1(x)$, and $\mathrm{E_d}(x)$, respectively, which agree well with the experimental results marked with symbols.

Supplementary Note 2: Additional results/discussion about pump-power dependence

In order to exclude the influence of pump lasers on the DFG signal and verify the difference-frequency relation during the DFG process, another two experiments are performed. The corresponding dispersion curves and the spectral information of the two new results are provided in Supplementary Fig. 2 and Supplementary Fig. 4, respectively.

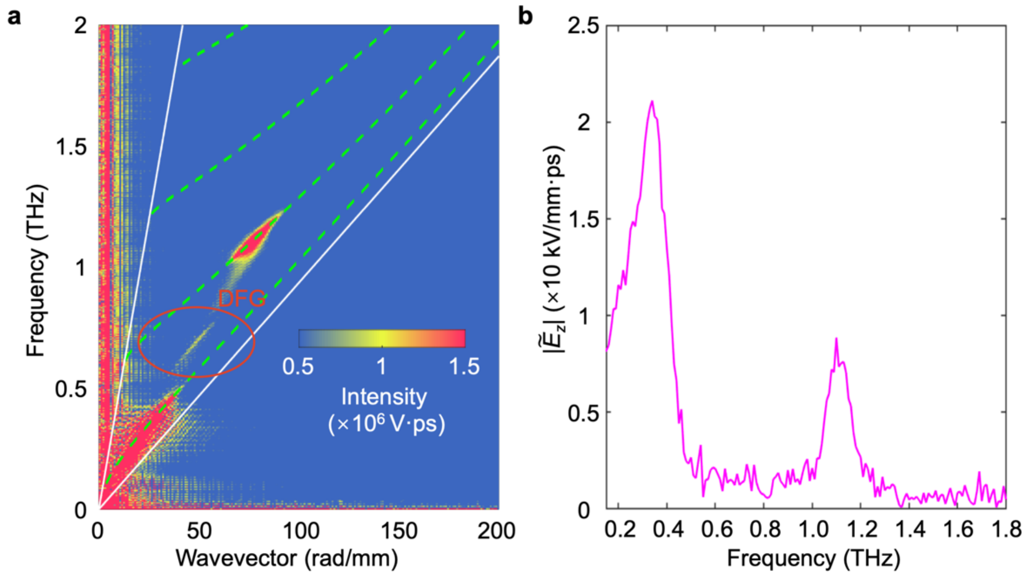

Supplementary Figure 2 Experiment under weak pump (about 81% smaller than the main-text value, marked as Experiment 1). **a** Experimental dispersion curve under a low pump power **b** The representative spectrum at the position x = 1.14 mm. The DFG signal can be hardly seen.

In these two results, the Experiment 1 is performed under a low-pump power (about 81% smaller than the main-text value), where the field amplitudes of the matched zero-order ($\nu_0' \approx \nu_0 = 0.34$ THz) and first-order ($\nu_1' \approx \nu_1 = 1.1$ THz) waveguide modes have similar frequencies as the results in the main text. However, the field amplitudes of the waveguide modes are much smaller than the values in the main text. In this case, the DFG signal is very weak and hardly to be seen in the spectra, as shown in Supplementary Fig. 2b. This result indicates that the DFG signal does depend on the two waveguide modes nonlinearly, and the nonlinear frequency-mixing process dominates the generation of E_d. Only in the dispersion curve can we see the weak DFG signal, as Supplementary Fig. 2a indicated, and a very small color-bar limit is used here in order to identify and distinguish the DFG signal. While the noise appears in the low-wavevector regime, thus the DFG signal is hardly seen in Supplementary Fig. 2b. Nevertheless, the noise causes little influence when the THz field is strong enough, as shown in Supplementary Fig. 3a and Supplementary Fig. 2c.

In order to further verify this result, we can calculate and evaluate the expected value amplitude of DFG signal according to the main-text nonlinear susceptibility of $\chi^{(2)} = 1.58$ mm/kV.

According to Supplementary Fig. 2b, we can calculate the field amplitude of E_0 and E_1 by a Gaussian fitting (the same method as in the main text), and we obtain

$$E_0(x = 1.14) = 0.74 \text{ kV/mm}$$

$$E_1(x = 1.14) = 0.22\ \mathrm{kV/mm}$$

By using the calculated nonlinear susceptibility, one can evaluate the DFG amplitude by

$$E_d = p\chi^{(2)}E_0E_1 \approx 0.1239\ \mathrm{kV/mm}$$

Here we ignored the propagation of E_d for simplicity. Suppose a temporal expansion of 4 ps (similar to the main-text experiment), we can obtain the relative intensity in the frequency domain.

After calculation, we can get the Fourier spectrum of the DFG signal, which is shown in Supplementary Fig. 3a. This value is indeed in the noise level of the Supplementary Fig. 2b, which is shown below as Supplementary Fig. 3b with superimposed data from Supplementary Fig. 2b for direct comparison (please notice that the two sub figures have different y-axis scales – differs by one order of magnitude).

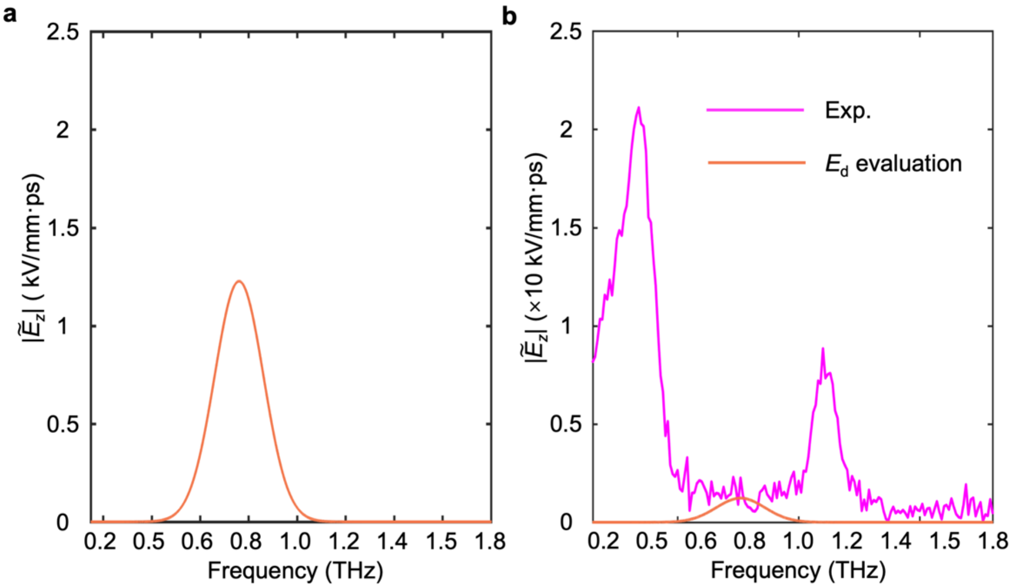

Supplementary Figure 3 The theoretical evaluation of the Fourier spectrum of difference frequency signal according to the main-text nonlinear susceptibility. a The Fourier spectrum of evaluated E_d. **b** The calculated E_d was superimposed onto the experimental data for comparison.

As Supplementary Fig. 4 shows, the Experiment 2 is performed under a high-pump power (about 43% larger than the main-text value), where the field amplitudes of the matched waveguide modes are larger than the values in the main text. In order to further verify the difference-frequency relation, we slightly change the wavefront tilt angle α. Then the zero-order $(\nu_0'' = 0.42\ \mathrm{THz} > \nu_0 = 0.34\ \mathrm{THz})$ and first-order $(\nu_1'' = 1.31\ \mathrm{THz} > \nu_1 = 1.1\ \mathrm{THz})$ have larger frequencies than the values in the main-text experiment, the DFG signal here also satisfies $\nu_d'' = \nu_1'' - \nu_0''$. Using the same method (gaussian-function fitting) in the main text, the fitting amplitudes of the signals at $x = 1.16$ mm and $x = 1.31$ mm are calculated to be:

$E(\nu_0'', x = 1.16)$ =6.216 kV/mm; $E(\nu_0'', x = 1.31)$ =6.382 kV/mm;

$E(\nu_1'', x = 1.16)$ =2.926 kV/mm; $E(\nu_1'', x = 1.31)$ =2.980 kV/mm;

$E(\nu_d'', x = 1.16)$ =2.129 kV/mm; $E(\nu_d'', x = 1.31)$ =1.682 kV/mm.

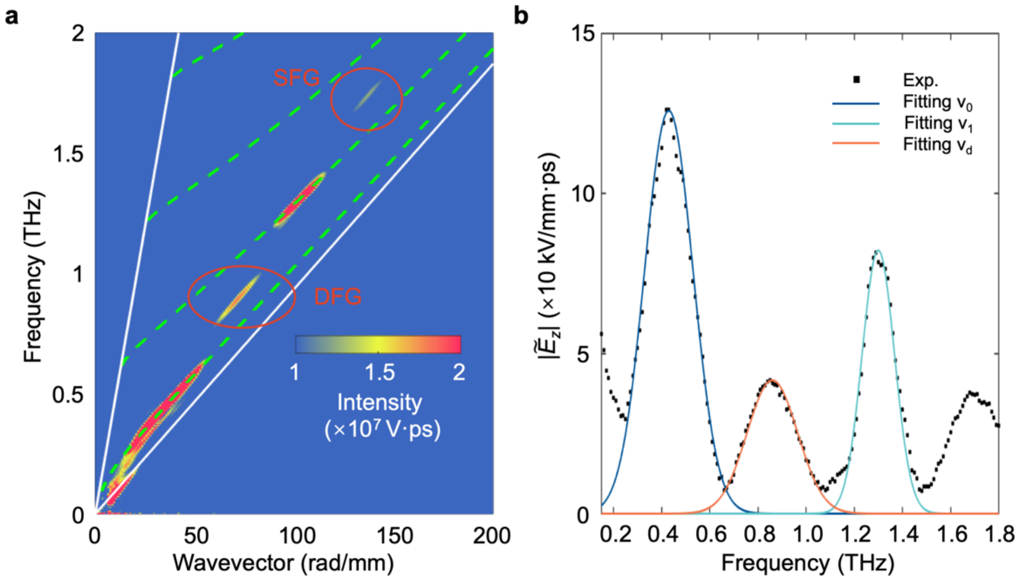

Supplementary Figure 4 Experiment under strong pump (about 43% larger than the main-text value, marked as Experiment 2). **a** Experimental dispersion curve under a high pump power (). The DFG and sum frequency generation (SFG) signals can be clearly seen. **b** The spectrum where the amplitudes of the DFG signal are large (x = 1.16 mm).

Considering the material absorption for THz waves at the frequencies ν_0'', ν_1'', and ν_d'' are $\alpha_0'' = 0.35$ mm^{-1}, $\alpha_1'' = 2.1$ mm^{-1}, and $\alpha_d'' = 1.1$ mm^{-1} respectively, we constructed a similar model as we used in the main text. After calculation, the nonlinear susceptibility shows a value of $\chi^{(2)} > 1.352$ mm/kV. Correspondingly, the theoretical value in these frequencies given by Eq. (4) in the main text is about 8.43 mm/kV. This result shows a good agreement with that in the main-text, although the nonlinear susceptibility and the material absorption are slightly different in higher frequencies.

In summary, we can conclude that the measured signal indeed comes from the DFG nonlinear process, rather than from the pump laser directly by the comparison of Experiment 1, Experiment 2, and the main-text experiment. Furthermore, in the theoretical view, the influence of pump laser on the DFG signals can also be excluded from the following aspects:

First, THz waves generated by pump laser (ISRS) have a broadband spectrum, which is centered at about 0.5 THz under the same condition, seen in our previous work[6]. However, in the tilted-wavefront generation, only the frequency components that match the waveguide modes remain, as shown in Fig. 4b, where even the energy distribution at about 0.5 THz is still very low. If the pump laser could generate THz waves at the difference frequency, it must also generate THz waves at the frequency range between the two waveguide modes, especially at 0.5 THz, rather than only at the difference frequency point.

Second, even if we assume ISRS could generate THz waves at the difference frequency point, it is different from the other two frequencies that match with the waveguide modes, where the generated THz waves could have a continual increase as a function of position x. Considering the general generation efficiency[5,6], a 400 μJ pump laser beam could generate THz waves with a maximum amplitude of 0.4 kV/mm focused by a cylindrical lens to a line of about 0.1 mm. Here we consider an extreme condition that the wavefront-tilted pump laser excites THz waves simultaneously in the $LiNbO_3$ waveguide, since the length of the pump laser on $LiNbO_3$ is

much larger (about 0.9 mm) than that in our previous work (about 0.1 mm), the field amplitude of the pump is about 1/9 of that value[6]. Accordingly, the THz intensity it generated is only $1/9^2$ of the value. Considering our practical setup, where the wavefront-tilted pump does not generate THz waves in a very short time span and for a very large waveguide mode attenuation, the practical THz waves at difference frequency point are far smaller, which is negligible compared with the DFG generated value.

Additionally, one can also see that the second harmonic generation (SHG) signal of ν_0 is absent in all of our experiments, while the second-order nonlinear susceptibility seems much larger than that in the previous reported results[7]. Here in our experiment, the different frequency component is selectively enhanced (compared with that from SHG) and the nonlinear susceptibility for the DFG process was calculated. The missing (or weak appearance at high pump power) of the SHG signal in our experiment is a complicated question, which could depend on several factors that include the phase-match selection, the transient effect, and the anisotropic subwavelength waveguide modes in the experiments. In addition, an unexpected enhancement of SFG signal at high-pump power case can also be observed. This puzzling behavior of the SFG in the high-pump power case seems to be caused by a false signal, since where the frequency of the SFG signal is nearly the resolution limit of our experimental system.

Supplementary Note3: Table

Supplementary Table 2 The comparison of second-order nonlinear susceptibility between our results and the common nonlinear optical materials. Asterisk marks the material with relevant parameters that have been used for THz wave generation[6].

DAST: (4-N, N-dimethylamino-4'-N'-methyl-stilbazolium tosylate);

DSTMS: (4-N, N-dimethylamino-4'-N'-methyl-stilbazolium-2,4,6-trimethylbenzenesulfonate);

OH1: (2-(3-(4-hydroxystyryl)-5,5-dimethylcyclohex-2-enylidene)malononitrile).

Materials	Frequency/Wavelength	$\chi^{(2)}, d$ (m/V)	References
ZnTe*	800 nm	$d = 6.6\times10^{-11}$	8,9
GaP*	1000 nm	$d = 2.4\times10^{-11}$	8,9
$LiNbO_3$*	1000 nm	$d = 1.6\times10^{-10}$	8,9
DAST*	1500 nm	$d = 2.4\times10^{-10}$	8,9
DSTMS*	1500 nm	$d = 2.3\times10^{-10}$	8
OH1*	1300 nm	$d = 2.8\times10^{-10}$	8
CdTe	1500 nm	$\chi^{(2)} = 1.035\times10^{-9}$	9-11
$AgGaSe_2$	10.6 μm	$d_{36}=3.95\times10^{-11}$	12
$ZnGeP_2$	9.6 μm	$d_{36}=7.5\times10^{-11}$	12
$CdGeAs_2$	10.6 μm	$d_{36}=1.86\times10^{-10}$	12
$HgGaS_4$	1.06 μm	$d_{36}=2.72\times10^{-11}$	12
$CdSiP_2$	4.56 μm	$d_{36}=8.45\times10^{-11}$	12
$AgGaTe_2$	10.6 μm	$d_{22}=7.5\times10^{-11}$	12
$LiGaTe_2$	4.5 μm	$d_{36}=4.3\times10^{-11}$	12
$AgGaS_2$	10.6 μm	$d_{36}=1.26\times10^{-11}$	12
$LiGaS_2$	2.3 μm	$d_{31}=5.8\times10^{-12}$	12
$LiGaSe_2$	2.3 μm	$d_{31}=9.9\times10^{-12}$	12
$LiInS_2$	2.3 μm	$d_{24}=7.7\times10^{-12}$	12
$LiInSe_2$	2.3 μm	$d_{31}=1.18\times10^{-11}$	12
$BaGa_4S_7$	2.3 μm	$d_{32}=5.7\times10^{-12}$	12
$BaGa_4Se_7$	2.3 μm	$d_{13}=-2.06\times10^{-11}$	12
Graphene (spatial dispersion)	3.1 μm	$0.3\sim3.5\times10^{-10}$	13
In:GaSe	THz	$\chi^{(2)} =(3.7\sim5.1)\times10^{-11}$	14
Ag:GaSe	THz	$\chi^{(2)} =7.5\times10^{-11}$	14
GaAs/AlGaAs quatun wells	THz	$\sim10^{-8}$	15
GaSe	THz	$d_{22}=(8.6\pm1.7)\times10^{-11}$	16
ZnTe	THz	$\chi^{(2)} =9\times10^{-11}$	17
Dark metasurface	THz	$\chi^{(2)} =2.4\times10^{-11}$	18
SRR metasurface	THz	$\chi^{(2)} =1.6\times10^{-12}$	18,19
$LiNbO_3$ (phonon polariton)	THz	$\chi^{(2)} =1.58\times10^{-6}$	This work

Supplementary References

1. Boyd, R. W. Nonlinear Optics. *Elsevier* (2003).
2. Yang, C. et al. Experimental and theoretical analysis of THz-frequency, direction-dependent, phonon polariton modes in a subwavelength, anisotropic slab waveguide. *Opt. Express* **18**, 26351 (2010).
3. Wu, X. et al. Temperature dependent refractive index and absorption coefficient of congruent lithium niobate crystals in the terahertz range. *Opt. Express* **23**, 29729 (2015).
4. Dougherty, T. P. et al. Femtosecond resolution of soft mode dynamics in structural phase transitions. *Science* **258**, 770-774 (1992).
5. Feurer, T. et al. Terahertz polaritonics. *Annu. Rev. Mater. Res.* **37**, 317 (2007).
6. Wu, Q. et al. Quantitative phase contrast imaging of THz electric fields in a dielectric waveguide. *Opt. Express* **17**, 9219 (2009).
7. Chen, Z., Zhou, X., Werley, C. A., & Nelson, K. A. Generation of high power tunable multicycle teraherz pulses. *Appl. Phys. Lett.* **99**, 071102 (2011).
8. Jazbinsek, M., Puc, U., Abina, A. & Zidansek, A. Organic Crystals for THz Photonics. *Applied Sciences-Basel* **9**, 982 (2019).
9. Hoffmann, M. C. & Fueloep, J. A. Intense ultrashort terahertz pulses: generation and applications. *Journal of Physics D: Applied Physics* **44**, 083001 (2011).
10. Rojan, K., Legar, Y., Morigi, G., Richard, M. & Minguzzi, A. Enhanced Second-Order Nonlinearity for THz Generation by Resonant Interaction of Exciton-Polariton Rabi Oscillations with Optical Phonons. *Phys. Rev. Lett.* **119**, 127401 (2017).
11. Jeon, Y. & Kang, H. S. Electro-optic coefficient measurements for ZnxCd1-xTe single crystals at 1550 nm wavelength. *Optical Review* **14**, 373-375 (2007).
12. Isaenko, L. I. & Yelisseyev, A. P. Recent studies of nonlinear chalcogenide crystals for the mid-IR. *Semiconductor Science and Technology* **31**, 123001 (2016).
13. Yao, X., Tokman, M. & Belyanin, A. Efficient Nonlinear Generation of THz Plasmons in Graphene and Topological Insulators. *Phys. Rev. Lett.* **112**, 055501 (2014).
14. Guo, J. et al. Doped GaSe crystals for laser frequency conversion. *Light: Science & Applications* **4**, e362 (2015).
15. Madeo, J. et al. All-optical wavelength shifting in a semiconductor laser using resonant nonlinearities. *Nature Photonics* **6**, 519-524 (2012).
16. Allakhverdiev, K. R., Yetis, M. O., Ozbek, S., Baykara, T. K. & Salaev, E. Y. Effective nonlinear GaSe crystal. Optical properties and applications. *Laser Physics* **19**, 1092-1104 (2009).
17. Shen, Y. et al. Nonlinear cross-phase modulation with intense single-cycle terahertz pulses. *Phys. Rev. Lett.* **99**, 043901 (2007).
18. Fang, M. et al. Nonlinearity in the Dark: Broadband Terahertz Generation with Extremely High Efficiency. *Phys. Rev. Lett.* **122**, 027401 (2019).
19. Luo, L. et al. Broadband terahertz generation from metamaterials. *Nat. Commun.* **5**, 1-6 (2014).
