# Supplementary material for: Giant enhancement of THz-frequency optical nonlinearity by phonon polariton in ionic crystals
Source: Nat Commun. 2021 May 26;12:3183. doi: 10.1038/s41467-021-23526-w (PMC8155090; doi:10.1038/s41467-021-23526-w)
Supplement: Supplementary file 3 — Description of Additional Supplementary Files [file 41467_2021_23526_MOESM3_ESM.pdf]

Description of Additional Supplementary Files

File name: Supplementary Movie 1

Description: The spatiotemporal evolution of THz difference frequency generation in lithium niobate waveguide.
